# The Association between Post-Migration Nutrition and Lifestyle Transition and the Risk of Developing Chronic Diseases among Sub-Saharan African Migrants: A Mixed Method Systematic Review Protocol

**DOI:** 10.3390/ijerph18094706

**Published:** 2021-04-28

**Authors:** Blessing Akombi-Inyang, Md. Nazmul Huda, Aletta E. Schutte, Rona Macniven, Sophia Lin, Patrick Rawstorne, Xiaoyue Xu, Andre Renzaho

**Affiliations:** 1 School of Population Health, Faculty of Medicine, University of New South Wales (UNSW), Sydney, NSW 2052, Australia; m.huda@unsw.edu.au (M.N.H.); a.schutte@unsw.edu.au (A.E.S.); r.macniven@unsw.edu.au (R.M.); sophia.lin@unsw.edu.au (S.L.); p.rawstorne@unsw.edu.au (P.R.); luna.xu@unsw.edu.au (X.X.); 2 The George Institute for Global Health, Sydney, NSW 2052, Australia; 3 Hypertension in Africa Research Team, Medical Research Council Unit for Hypertension and Cardiovascular Disease, North-West University, Potchefstroom 2520, South Africa; 4 Translational Health Research Institute (THRI), Western Sydney University, Sydney, NSW 2052, Australia; Andre.Renzaho@westernsydney.edu.au; 5 School of Medicine, Western Sydney University, Penrith, NSW 2751, Australia; 6 Maternal, Child and Adolescent Health Program, Burnet Institute, Melbourne, NSW 3004, Australia

**Keywords:** diet, malnutrition, transition, non-communicable diseases, obesity, cardiovascular diseases, Sub-Saharan Africa, migration, acculturation

## Abstract

Sub-Saharan African (SSA) migrants face nutrition and lifestyle changes upon arrival in a host country. The shift in diet and lifestyle reflects post-migration acculturation and could predispose migrants to nutrition- and lifestyle- related chronic diseases. A mixed-methods systematic review of published studies and the grey literature on post-migration nutrition and lifestyle transition among SSA migrants will be undertaken. Studies published in English and conducted from 2000 to 2020 using quantitative and/or qualitative methods will be included. Ten bibliographic databases will be searched: Scopus, Ovid MEDLINE, EMBASE, Global Health, CINAHL, PubMed, ProQuest, PsycINFO, Informit and Web of Science. Data extraction will be informed by the Cochrane PROGRESS-Plus framework and the Joanna Briggs Institute manual. The quality of the included studies will be appraised for risk of bias using validated tools. An integrated approach to quantitative and qualitative data synthesis through data transformation will be undertaken, and a narrative synthesis of the findings will be provided. This protocol is guided by the Preferred Reporting Items for Systematic Reviews and Meta-Analyses Protocols (PRISMA-P) guidelines and provides insight into the scope and parameters of the systematic review to be conducted. The aim of the review is to evaluate the association between post-migration nutrition and lifestyle transition and the risk of developing chronic diseases among SSA migrants in Australia. This review will provide insight into possible areas for interventions to improve the health of migrants. *Systematic Review Registration: The protocol was registered with the PROSPERO international prospective register of systematic reviews* CRD42020206560.

## 1. Introduction

Australia has a large culturally and linguistically diverse (CALD) community [1] with more than a quarter of its population born overseas [2]. Of these, 5.6% are from Sub-Saharan Africa (SSA), constituting about 1.3% of the total Australian population with the largest SSA cohorts coming from South Africa (162,449), Zimbabwe (34,787), Mauritius (24,329), Kenya (17,652), Ethiopia (11,792), Nigeria (8488), Somalia (7668), Zambia (6235) and Ghana (5287) [2,3].

SSA migrants typically benefit from good health on arrival due to the stringent medical examination that migrants are subject to before being granted entry into Australia [4]. This places these migrants at a mortality and morbidity advantage relative to most of the population in Australia—a phenomenon called the healthy migrant effect [5,6,7]. However, a few years post-migration, the healthy migrant effect appears to diminish, and suboptimal nutritional status tends to be observed [7,8,9].

The determinants of overweight, obesity and micronutrient deficiencies, including iron deficiency anaemia and vitamin D deficiency, among SSA migrants are manifold, and the outcome of their impact tends to increase significantly after 10–15 years of migration [10,11]. With a shift in dietary consumption and lifestyle due to a change in environment [12], SSA migrants become more susceptible to nutrition- and lifestyle-related chronic diseases, such as cardiovascular diseases, hypertension, diabetes, cancer, osteoporosis, and dental diseases [7]. The complex relationship between nutrition and lifestyle transition and chronic diseases can be explained from a theoretical perspective which considers the influences of acculturation [12,13,14]. The process of acculturation does not follow a linear process; rather, it is a bi-dimensional model of the strength of cultural affiliation that may lead to four cultural orientations: traditional (origin culture), assimilated (host culture), integrated (both origin and host culture) and marginalized (neither origin nor host culture) [15]. This cultural adaptation and immersion of SSA migrants over time [15,16,17] and the rising uptake of obesogenic behaviours characterised by the adoption of high-calorie diets and sedentary behaviours which are common in many developed countries [7,15] are indicative of the broader process of acculturation where migrants assimilate the host culture [18,19,20]. Research has shown that the drivers of this rapid increased risk are mainly socio-cultural norms and values around body sizes and exacerbated by acquired negative dietary habits (dietary acculturation) and poor health literacy [21]. This phenomenon has consistently been associated with nutrition- and lifestyle-related chronic diseases among migrants [7,19,22] but no reviews have focused on SSA migrants in Australia. Therefore, this mixed-methods review will highlight the association between post-migration nutrition and lifestyle transition and the risk of developing chronic diseases among SSA migrants in Australia to inform future interventions to reduce the rate of chronic diseases.

### Aim of the Review and Its Public Health Importance

This review will identify, collate, synthesise, and appraise the available literature on the association between post-migration nutrition and lifestyle transition and the risk of developing chronic diseases among SSA migrants. Previous studies have focused on the dietary patterns and lifestyle behaviours of Australians and the risk of developing chronic diseases [23,24,25,26,27,28,29,30,31]. However, no review has synthesised evidence on the relationship between a shift in nutrition and lifestyle post-migration and the risk of lifestyle-related chronic diseases among SSA migrant populations in Australia. Understanding the health implications of nutrition and lifestyle transition will inform community-based interventions, policy, and structural reforms to improve health outcomes among SSA migrants. This proposed review will collate evidence on the health impacts of post-migration nutrition and lifestyle transition among SSA migrants in Australia. It will summarise evidence that considers the health implications of acculturation in SSA migrants. It aims to contribute to the existing evidence needed to inform interventions strategies and prioritise actions on improving health literacy among SSA migrants in Australia.

## 2. Materials and Methods

### 2.1. Study Design

This protocol is reported in accordance with the standard Preferred Reporting Items for Systematic Reviews and Meta-Analyses Protocols (PRISMA-P) guidelines [32] (Supplementary file S1). Studies in the review will include peer-reviewed quantitative, qualitative and mixed-methods studies. The grey literature, including research reports, books and book chapters, conference papers and proceedings, working papers, programme and evaluation reports, government documents, thesis and dissertations, will also be included.

### 2.2. Outcomes of Interest

The primary outcome will be nutrition- and lifestyle-related chronic diseases, including cardiovascular diseases, diabetes, hypertension, cancer, osteoporosis, dental diseases, and obesity.

### 2.3. Participants

The target population will be SSA migrants residing in Australia, as categorised by the International Organisation for Migration [33].

### 2.4. Inclusion and Exclusion Criteria

Studies will be included in the review if they (1) report the relationship between post-migration changes in nutrition habits and lifestyles and nutrition- and lifestyle-related chronic diseases; (2) are peer-reviewed quantitative and/or qualitative studies—the inclusion of both quantitative and/or qualitative methods will enable the triangulation of findings, thus increasing the credibility and validity of the findings [34]; (3) are grey literature—the inclusion of grey literature in this review is useful in complementing study findings from the peer-reviewed literature; (4) were published in English between January 2000 and September 2020 with full texts available and accessible. The research team is unable to retrieve and translate articles not published in English due to a lack of financial and logistical capacity. The year 2000 was chosen as the base year due to the rapid rise in migration globally, with Oceania—which includes Australia—reporting an approximate 31 percent population growth [35].

Studies will be excluded if they (1) do not report a relationship between post-migration changes in nutrition habits and lifestyles and nutrition- and lifestyle-related chronic diseases; (2) do not focus on SSA migrants in Australia; (3) are commentaries, reviews, opinion pieces, study protocols, letters to editors and editorials; (4) are not published in English.

### 2.5. Search Strategy

A list of relevant keywords and search terms will be generated and used to extensively search ten bibliographic databases, including Scopus, Ovid MEDLINE, EMBASE, Global Health, CINAHL, PubMed, ProQuest, PsycINFO, Infomit and Web of Science. In the case of relevant publications which might be missed during the initial search, a further search of the bibliographical references of all eligible publications, complemented by citation tracking using Google Scholar, will be conducted.

In this review, subject heading truncations (*), and Boolean operators (“AND”, “OR” and “NOT”) will be applied depending on the specifications of databases to be searched. A research librarian was consulted to finalise the search strategy. The search syntax has been tested and yielded a manageable number of records. Database searches will be re-run prior to the final analysis.

### 2.6. Study Selection

Studies identified in the search will be exported into an EndNote library and duplicates will be removed. Study selection will be carried out in three phases. The first phase will involve the screening of titles for relevance. In the second phase, the screening of abstracts will be conducted for eligibility and relevance. Finally, the full text of the retained studies will be further reviewed for final inclusion. The study selection will be carried out by two independent researchers (BA and MNH), and all disagreements between the two researchers will be resolved through discussion and consensus. In the case where a consensus is not reached, a third researcher (AES) will be called to adjudicate eligibility.

## 3. Results

### 3.1. Data Extraction

Data extraction will be informed by the PRISMA guidelines [32]. A piloted form based on the PROGRESS-Plus framework [36] will be used for data extraction. A growing number of systematic reviews utilise this framework [37,38,39,40,41] to study health inequities and evaluate interventions among different populations including migrants [41]. The data extracted will include author(s), publication year, study design and setting, sampling and data collection method(s), population characteristics, intervention, outcomes, and quality assessment. This process will be carried out by two independent researchers (BA and MNH), a third researcher (AR) will resolve any disagreements.

### 3.2. Data Synthesis

Due to the heterogeneity and variation in methodology and outcomes between the studies, a statistical aggregation of the data may not be appropriate. However, to develop a robust understanding of the association between post-migration nutrition and lifestyle transition and the risk of developing lifestyle-related chronic diseases, an integrated approach to quantitative and qualitative data synthesis through data transformation will be undertaken [42]. This approach involves extracting common threads from quantitative narratives and qualitative themes and modifying the format to provide a deeper understanding into post-migration nutrition and lifestyle transition among SSA migrants. The study findings will be summarised, and emerging themes narrated.

### 3.3. Quality Assessment

Prior to final inclusion in the review, an assessment of the methodological quality of eligible studies will be conducted to evaluate (i) internal validity—based on potential measurement biases and confounding; (ii) external validity—based on potential selection bias. The methodological quality of eligible studies will be assessed using the Critical Appraisal Skills Programme (CASP) for qualitative studies [43]; randomised controlled trials [44]; and observational studies, including longitudinal studies [45] and case–control studies [46]. The Mixed-Methods Appraisal Tool (MMAT) by Pluye and colleagues [47] will be used to assess mixed-methods studies. The grey literature will be assessed using the authority, accuracy, coverage, objectivity, date, and significance (AACODS) tool [48]. Eligible studies will be appraised as either high, medium, or low quality, and the Grading of Recommendations Assessment, Development and Evaluation (GRADE) approach [49] will be used to assess the overall quality of eligible studies included in the review. The quality assessment process will be carried out by two independent researchers (BA and MNH), and any disagreements will be resolved through discussions. In the case where an agreement is not reached between the two researchers, a third researcher (AR) will independently review the evidence and adjudicate.

## 4. Discussion

There is growing interest among governments, donor communities and philanthropic and community development planners in Australia to understand and promote nutrition and lifestyle-related interventions to minimise the risk of chronic diseases among SSA migrants [7]. The susceptibility of SSA migrants to chronic diseases and the necessity for nutrition and lifestyle-related interventions highlight a research and policy priority. A substantial pool of literature exists on the subject matter but remains unsynthesised. The available literature necessitates the need for a review which provides a robust summary of evidence to influence policies related to nutrition and lifestyle transition among SSA migrants. The findings of the review will inform the design of nutrition and lifestyle-related interventions to reduce chronic diseases among SSA migrants in Australia.

## 5. Conclusions

The proposed review will collate and synthesise evidence on the health impacts of post-migration nutrition and lifestyle transition among SSA migrants in Australia as well as provide insight into possible areas for interventions to improve migrant health within Australia and globally.

## Supplementary Materials

The following are available online at https://www.mdpi.com/article/10.3390/ijerph18094706/s1, Supplementary file S1: PRISMA-P checklist.
